# Validation of a constraint-based model of *Pichia pastoris *metabolism under data scarcity

**DOI:** 10.1186/1752-0509-4-115

**Published:** 2010-08-17

**Authors:** Marta Tortajada, Francisco Llaneras, Jesús Picó

**Affiliations:** 1 Biopolis S.L., C/Catedrático Agustín Escardino Benlloch, 9, 46980, Paterna, Valencia, Spain; 2 AI2, Universidad Politécnica de Valencia, Camino de Vera s/n, 46022, Valencia, Spain

## Abstract

**Background:**

Constraint-based models enable structured cellular representations in which intracellular kinetics are circumvented. These models, combined with experimental data, are useful analytical tools to estimate the state exhibited (the phenotype) by the cells at given pseudo-steady conditions.

**Results:**

In this contribution, a simplified constraint-based stoichiometric model of the metabolism of the yeast *Pichia pastoris*, a workhorse for heterologous protein expression, is validated against several experimental available datasets. Firstly, maximum theoretical growth yields are calculated and compared to the experimental ones. Secondly, possibility theory is applied to quantify the consistency between model and measurements. Finally, the biomass growth rate is excluded from the datasets and its prediction used to exemplify the capability of the model to calculate non-measured fluxes.

**Conclusions:**

This contribution shows how a small-sized network can be assessed following a rational, quantitative procedure even when measurements are scarce and imprecise. This approach is particularly useful in lacking data scenarios.

## Background

The collection of biochemical reactions involved in the metabolism of a cell can be assembled in networks in order to carry out studies under a system-level approach [1]. Such analysis have been done with large, even genome-scale, reconstructions of well-characterised organisms such as *Escherichia coli*, *Saccharomyces cerevisiae, Pseudomonas putida *[2-4], and also with simpler networks that consider only a few key metabolites [5-7].

Given a metabolic network, a matrix equation can be used in order to describe the mass balances around the nodes, the *m *internal metabolites:

(1) $$\frac{\text{d}c}{\text{dt}}=S\cdot v$$

in which **c **is a vector of metabolite concentrations and **v **is the vector of reaction rates, or fluxes, representing the mass flow through each of the *n *reactions in the network [8].

In order to avoid reaction kinetics, still rarely known, the internal metabolites are often assumed not to accumulate and thus (1) turns into a system of linear equations. Then, other constraints can be imposed; for instance, it is common to consider particular enzyme kinetics [9], thermodynamics [2,10], or the irreversibility of certain reactions using inequalities. In this way, a constraint-based model can be assembled [11,12].

By combination of this model and a set of measurable fluxes, the remaining ones can be estimated performing a metabolic flux analysis (MFA) [13]. It is even possible to incorporate intracellular measurements from stable isotope tracer experiments to apply 13C-MFA [14,15]. Unfortunately, these data are not available in most cases. Indeed, scarcity of measurements often results in practice in underdetermined systems, and therefore traditional MFA cannot be performed. In this context, a constraint-based approach that attempts to provide a range of candidate flux states instead of predicting the actual one with precision [11,16] can be of use. In any case, MFA can only be performed using reasonably small networks with favourable structures: otherwise its under-determinacy can be neither removed, even when tracer experiments are available, nor reduced enough to get valuable estimates with a constraint-based approach.

Besides, these medium-sized networks are derived from the known biochemical reactions involved in the metabolism of a cell, and rely necessarily on reductionist hypothesis, being their validation often insufficient. They are seldom validated against datasets different from the one of interest, which is thus inconveniently used both to validate the model and to perform the MFA analysis. Herein we discuss a procedure seeking for further validation of these networks.

The methylotrophic yeast *Pichia pastoris *is world-wide recognized as a reference platform for the expression of recombinant proteins in eukaryotes, due to the possibility to grow cultures to very high cell densities, its ability to produce post-translational modifications, and the good protein yield/cost ratio. Heterologous genes are cloned under *P. pastoris *strong and tightly regulated alcohol oxidase promoter, and thus expressed when the cells grow on methanol as sole or combined carbon source.

The optimization of recombinant protein expression in *P. pastoris *has been usually addressed heuristically. Only a few publications [17-19] describe rational, model-based optimisation and control of *Pichia *growth and protein production. Among these, semi-structured, metabolism-based models representing intracellular behaviour are particularly rare [20,21].

In the following sections, a constraint-based model of *P. pastoris *will be described and validated against the available experimental data. Then, its ability to predict non-measured fluxes will be illustrated by estimating the biomass growth rate. The potential use of the model for the estimation of intracellular fluxes will also be discussed. In summary, this work applies a systematic, yet simple, procedure to provide further validation for a small-sized model of *P. pastoris*, using only data from extracellular measurements.

## Methods

### Constraint-based model

A constraint-based model, assuming that internal metabolites are at steady-state and considering the irreversibility of some reactions, can be described with a set of model constraints (ℳOC) as follows:

(2) $$ℳOC=\{\;\begin{array}{c} N\cdot v=0\\ D\cdot v\geq 0 \end{array}$$

Where **v **is the vector of reaction rates, or fluxes, representing the mass flow through each of the *n *reactions in the network, N is the stoichiometric matrix, and **D **is a diagonal matrix with **D**_**ii **_= 1 if the flux *i *is irreversible (otherwise 0).

The constraints in (2) define a space of feasible steady-state flux distributions, or flux states, which ideally comprises every theoretically possible phenotype: only flux vectors **v **that fulfill (2) are considered valid cellular states.

### Consistency analysis

The simplest consistency analysis could be performed checking that the flux states shown by cells fulfill the constraints imposed by the model. However, this simple approach would be impractical because measurements are imprecise and do not *exactly *satisfy the constraints. Such difficulty is overcome by taking into account uncertainty, as follows:

(3) $$w_{m}=v_{m}+e_{m}$$

where **e**_**m **_represents the error or deviation between the actual fluxes **v**_**m **_and the measured values **w**_**m**_.

Model and measurements can be consistent if there is a vector **v **fulfilling (2) and (3) for "reasonably small" deviations **e**_**m**_. Otherwise, we will conclude that model and measurements are inconsistent. An easy way to evaluate consistency is to find the flux vector **v **fulfilling (2) and (3) that minimises the (variance-weighted) sum of errors:

(4) $$\begin{array}{cccc} \min & \Phi =e_{m}^{T}\cdot F^{-1}\cdot e_{m} & \;s.t. & ℳOC \end{array}$$

Where it is assumed that **e**_**m **_are distributed normally with zero mean value and have a variance-covariance matrix **F**. If only linear equality constraints are considered in ℳOC, the residual ϕ is a stochastic variable following a **χ**^2^-distribution, and therefore a **χ**^2^-test can be used to detect and evaluate the inconsistency. The **χ**^2^-test is based upon statistical hypothesis testing to determine if the deviation is within expected experimental error [8]. However, we want to consider inequality constraints in (2), and therefore the **χ**^2^-test cannot be used because its assumptions are not fulfilled (ϕ does not follows a **χ**^2^-distribution anymore). Yet, the residual ϕ provides at least a rough indication of consistency.

### Consistency analysis: Possibilistic MFA

The consistency analysis can also be formulated as a possibilistic constraint satisfaction problem, as it has been recently proposed in [16]. The basic idea is that a flux vector fulfilling the model constraints (2) and compatible with the measurements will be considered "possible", otherwise "impossible". This can be refined to cope with measurements errors by introducing the notion of "degree of possibility".

We introduce a set of measurements constraints (ℳℰC) considering imprecision, as in (3), but substituting **e**_**m **_by two pairs of non-negative decision variables (non-negative variables are chosen to formulate the calculations as linear programming problems [16]):

(5) $$ℳℰC=\{\begin{array}{c} w_{m}=v_{m}+\varepsilon_{1}-\mu_{1}+\varepsilon_{2}-\mu_{2}\\ \varepsilon_{1},\mu_{1}\geq 0\\ 0\leq \varepsilon_{2}\leq \varepsilon_{2}^{\text{max}}\\ 0\leq \mu_{2}\leq \mu_{2}^{max} \end{array}$$

These decision variables *ε*_1_, *μ*_1_, *ε*_2 _and *μ*_2 _relax the basic assertion **w**_**m **_= **v**_**m**_, conforming a set of possibility distributions in (*w*_*m*_, *v*_*m*_) associated to some cost index J. Among different possible choices, a simple -yet sensible- one is the linear cost index:

(6) $$\text{J}=\alpha \cdot \varepsilon_{1}+\beta \cdot \mu_{1}$$

with *α*≥0 and *β*≥0, which are row vectors of measurement reliability coefficients.

The possibility π of each solution δ of (2) and (5), which corresponds to a particular flux vector **v**, is given by the value of the cost index:

(7) $$\begin{array}{cc} \pi (\delta )={\text{e}}^{-\text{J}(\delta )} & \delta \in ℳℰC\cap ℳOC \end{array}$$

The interpretation of (5-7) may be: "**w**_**m **_= **v**_**m **_is fully possible; the more **w**_**m **_differs from **v**_**m**_, the less possible such situation is". See the article for further technical details [16].

Defining two pairs of decision variables, there is more flexibility to represent the measurements in possibilistic terms: the user can assign the bounds ε_2_^*max *^and μ_2_^*max *^and the weights *α *and *β*. This way, each measurement is represented by a distribution of possibility (see examples in [16]). The bounds ε_2_^*max *^and μ_2_^*max *^define an interval of fully possible values (possibility π = 1). For instance, the user can choose a band of 10% around the measured value. The values *α *and *β *define the decreasing possibility to assign to values out of this interval (details below).

At this point, the maximum possibility (minimum-cost) flux vector **v**_**mp **_corresponding to a given set of measurements is obtained solving a linear programming (LP) problem:

(8) $$\begin{array}{ccc} \underset{\varepsilon ,\mu ,\text{v}}{\min} & \text{J} & s.t.\left\{ \begin{array}{l} ℳOC\\ ℳℰC \end{array}\right. \end{array}\;$$

The possibility of the most possible solution being, $\pi_{\text{mp}}=\pi (v_{mp})={\text{e}}^{-{\text{J}}^{\max}}$.

This degree of possibility provides an indication of the consistency between model (ℳOC) and measurements (ℳℰC): a possibility equal to one must be interpreted as complete agreement between the model and the original measurements; lower values of possibility imply that certain error in the measurements is needed to find a flux vector fulfilling the model constraints.

### Possibilistic estimation of non-measured fluxes

Possibilistic MFA also enables estimating the metabolic fluxes based on the model and the available measurements. The simplest point-wise estimate is the minimum-cost flux vector resulting from (7), which contains the most possible value for each flux. However, a point-wise estimate is limited when multiple combinations might be reasonably possible. In this situation, a possibilistic interval estimate is a better choice.

The interval of values with conditional possibility higher than for a given variable, $\left[{\text{v}}_{\text{i},\gamma }^{\text{m}},{\text{v}}_{\text{i},\gamma }^{\text{M}}\right]$, can be computed solving two LP problems,

(9) $$\begin{array}{ccc} v_{i,g}^{m}=\underset{{}_{\varepsilon ,\mu ,v}}{\min} & v_{i} & \text{s}.\text{t}.\{\begin{array}{c} ℳOC\cap ℳℰC\\ \text{J}-\log\pi (v_{m})<-\log\gamma \end{array} \end{array}$$

The upper bound ${\text{v}}_{\text{i},\gamma }^{\text{M}}$ would be obtained by replacing minimum by maximum. Possibilistic intervals have a similar interpretation to "confidence intervals" ("credible intervals") in Bayesian statistics, and provide concise but rich flux estimates. Please refer to the above-mentioned article for details on the possibilistic framework [16].

## Results and Discussion

### Metabolic Network of *P. pastoris*

**Figure 1:**
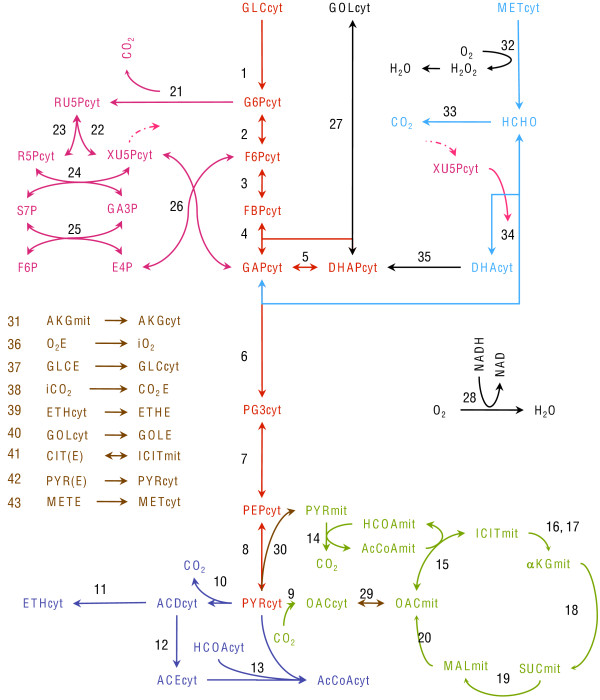
**Metabolic network of *P. pastoris***. Simplified representation of central carbon metabolism of the yeast during growth on glucose, glycerol and methanol. A supplementary reaction represents biomass formation from selected metabolites (see Additional File 1).

The metabolic network presented in Figure 1 is based on the stoichiometric model defined in [22] for *P. pastoris *growth on glucose, which has been extended with reactions representing methanol and glycerol metabolism. This is a simplified representation whose objective is not to accurately describe the full biochemistry of the yeast but to generate a model in which to apply methodologies of interest aimed to process analysis, monitoring and control.

The main catabolic pathways of the yeast *P. pastoris *(Embden-Meyerhoff-Parnas pathway, citric acid cycle, pentose phosphate and fermentative pathways) are represented for growth on the substrates mainly used for its culture: glucose, glycerol and methanol. In this case, a mean biomass equation derived from the macromolecular composition of the yeast is used to summarize the anabolic pathways according to [22]. Key metabolites such as NAD, NADP, AcCoA, oxalacetate and pyruvate are considered in distinct cytosolic and mitochondrial pools. Several alternative biomass equations corresponding to *Saccharomyces cerevisiae *models coming from the literature [4,23,24] were also tested (data not shown) as detailed in the following sections, and found to provide similar results. However, it would be useful to evaluate the sensitivity with particularized *P. pastoris *biomass compositions, if available.

The model contains 45 compounds and 44 metabolic reactions. The balanced growth condition can be applied to 36 internal metabolites, resulting in a 36 × 44 stoichiometric matrix with 8 degrees of freedom (the matrix and the list of reactions is given in the additional file 1). As in [22], irreversibility is assumed for all reactions except for {2-8; 15; 22-27; 29; 34}, and reaction 41 in order to account for glycerol uptake, resulting in the constraint-based model of the form (1), which is used hereinafter.

### Elementary mode analysis

Elementary mode analysis provides a way to systematically identify a set of relevant pathways of a metabolic network [25-27]. The elementary modes (EM) are the simplest (steady-state) flux distribution that cells can show, whereas the remaining feasible states can be seen as its aggregated action (without cancelations of reversible fluxes). Moreover, the fact that they comprise all the simple pathways in the network, the functional states or non-decomposable vectors, makes it possible to investigate the infinite behaviours that cells can show by simply inspecting them. They have been used, for instance, to analyse pathways considering optimality [25,28], determine minimal medium requirements [12], and infer viability of mutants [29].

**Figure 2:**
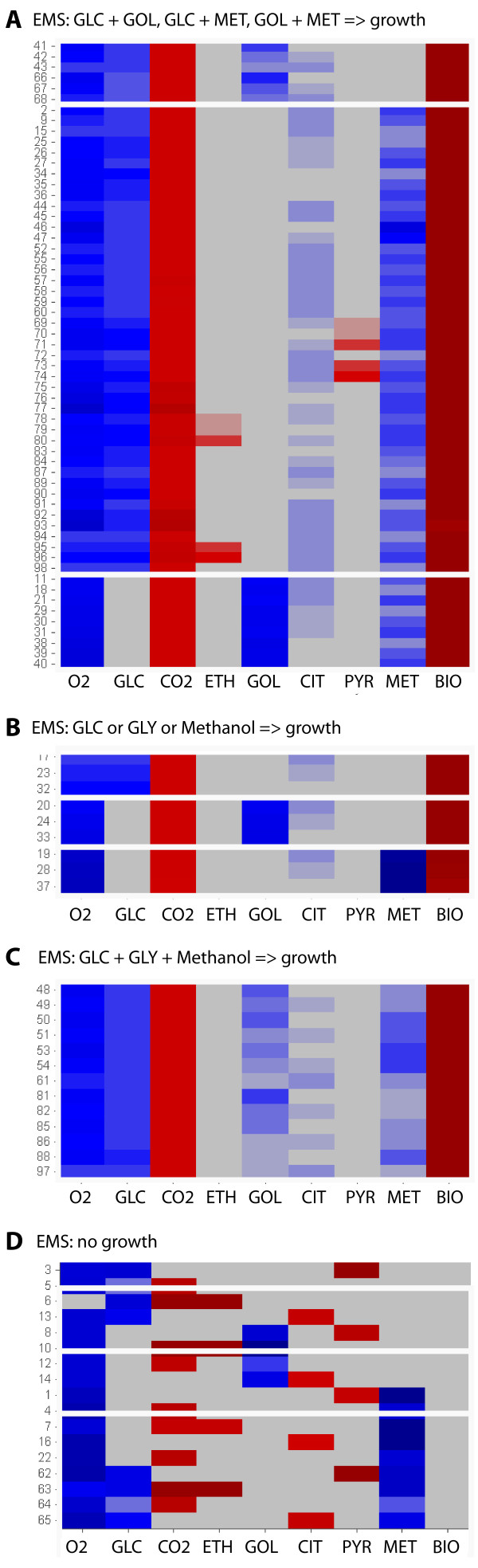
**Elementary modes of the network of *P. pastoris***. Macroscopic equivalents of the corresponding elementary modes. Blue denotes substances being consumed by the EM, and red those being produced (the darker, the higher stoichiometric coefficient). Arrows highlighted those EMs with the maximum theoretical yield (in terms of growth) for each type.

The 98 elementary modes for the described network were obtained using Metatool [30]. They are given in the additional file 2. The set of EMs can be classified as shown in Figure 2 depending first on its ability to produce biomass, and second on the carbon source used: glucose, methanol or glycerol. There are 17 EMs that do not result in biomass production, whereas 9 generate ethanol. No ethanol is produced in single substrate EMs when growing.

The carbon yields for biomass obtained for each EM as shown in Table 1. The maximum yield is 4.93 Cmol dcw/Cmol in presence of glucose. Glucose is the most efficient substrate for growth also in combination with glycerol or methanol.

**Table 1:** Maximal Yields (Cmol DW mol^-1^)

Glu	Glyc	Met	YTotal	EM
x			4.93	32
	x		2.46	33
		x	0.82	37
x	x		3.68	41
	x	x	2.25	38
x		x	3.98	34
x	x	x	3.47	85

**Figure 3:**
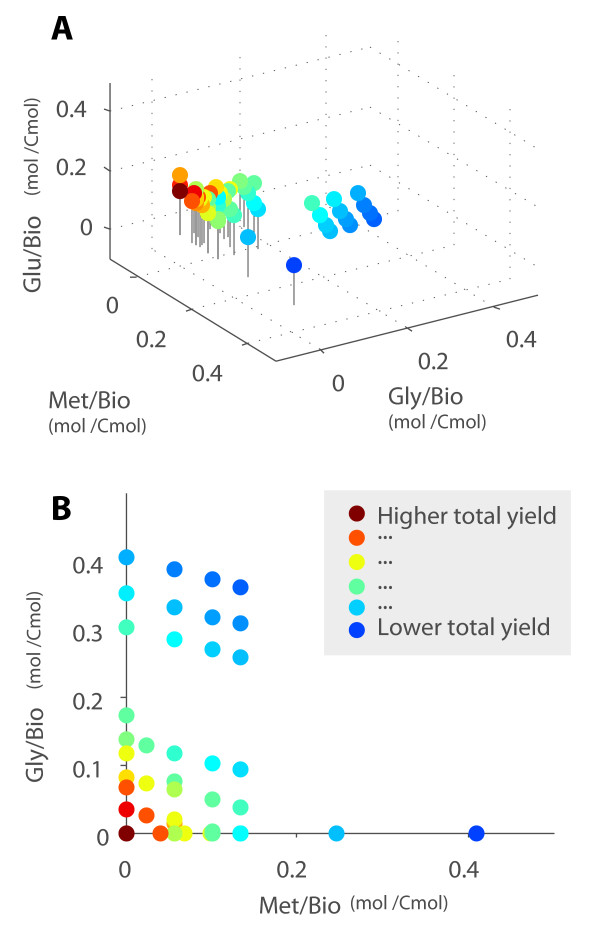
**Biomass yields for the Elementary Modes**. Panel A represents in each axis single substrate consumption for biomass growth. Most efficient modes are located nearer to origin. Panel B details frontal projection for growth on glycerol and methanol. The most profitable EM are glucose-consuming.

Methanol is the worst biomass yielding substrate. This is also illustrated in Figure 3. In the following sections 11 different datasets compiled from the literature (Table 2) are used to determine whether the simplified model described above is coherent with experimental data.

**Table 2:** Experimental data and model consistency

Ref*	μ	**Q**_**Glu**_	**Q**_**Gly**_	**Q**_**Met**_	**Q**_**et**_	OUR	CPR	**Q**_**P**_	**Yields Exp./Theo**.		Consistency**		
		
	**Cmol·kg**^**-1**^**·h**^**-1**^	**mol·kg**^**-1**^**·h**^**-1**^	"	"	"	"	"	**mg·g**^**-1**^**·h**^**-1**^	Cmol DW/mol	"	ϕ	π	To π = 1
		
D1	3.86	0.97	0.00	0.00	0.00	2.02	2.07	0.020	3.98	< 6.62	0.03	1.00	2%
A1	1.88	0.00	1.09	0.00	0.00	2.16	1.56	0.000	1.73	< 2.46	0.28	1.00	7%
A2	2.07	0.00	0.95	0.63	0.00	2.70	1.70	0.001	1.31	< 2.25	1.20	0.73	12%
A3	1.72	0.00	0.74	1.48	0.00	3.90	2.10	0.014	0.77	< 2.25	2.81	0.25	20%
A4	2.02	0.00	0.57	2.33	0.00	4.85	2.21	0.024	0.70	< 2.25	5.36	0.09	29%

B1	6.17	0.00	2.75	0.00	0.00	3.62	2.35	0.000	2.24	< 2.46	0.07	1.00	4%
B2	6.18	0.00	2.77	1.87	0.00	7.19	4.18	0.001	1.51	< 2.25	0.88	0.82	12%
B3	6.24	0.00	2.23	2.73	0.00	7.20	3.60	0.012	1.26	< 2.25	2.34	0.32	19%

C1	2.32	0.00	0.67	2.01	0.00	3.21	1.77	0.012	0.78	< 2.25	0.15	1.00	5%
C2	2.32	0.00	0.28	3.18	0.00	3.79	2.09	0.021	0.63	< 2.25	0.74	1.00	10%
C3	2.32	0.00	0.00	4.02	0.00	4.22	2.33	0.022	0.52	< 0.82	1.55 > 10	0.49	15%
Random	0-10	0-10	0-10	0-10	0-10	0-10	0-10	-			99% > 10	< 0.1 99%	-
Random	1.5-6	0-2	0-2.7	0-2.7	0-0.1	2.1-7.2	1.5-4	-			86%	< 0.1 95%	-

*All the datasets correspond to continuous fermentations in defined chemical media. Further detail can be found in D: Dragosits et al. [22]; A: Solà et al. [21]; B: Solà et al. [21]; C: Jungo et al. [19]. Citrate and Pyruvate are assumed not to be produced nor consumed except for dataset D1 in which citrate is consumed at 0.007 Cmol·kg^-1·^h^-1^.

**Minimized sum of squared residuals (ϕ), possibility of the most possible flux vector (P) and degree of measurements uncertainty to P = 1.

### Validation: experimental and theoretical yields

As a first validation, we checked that the experimental growth yields did not exceed the maximum theoretical ones given by the model (which were obtained by inspection of the elementary modes on each category). For instance, the theoretical yield for growth on glucose is 4.93, whereas the experimental one is 3.98 (Cmmol DW/mmol). The maximum yield on glycerol and methanol is 2.25, and the experimental ones at different ratios of glycerol and methanol range between 1.31 and 0.63. It also seems that the experimental yields decrease for combinations of substrates with lower theoretical yields.

Thus, no experimental yield violates the maximum theoretical ones (the contrary would indicate errors in the model because theoretical yields were obtained from it). However, the experimental yields tend to be lower than theoretical ones. There are several reasons for this deviation: (a) the model does not consider restrictions on energy cofactors, such as ATP, nor the resources devoted to recombinant protein production, (b) the EM analysis does not take into account the ratio between the different substrates in mixed cases, and (c) even if optimal pathways exist, the actual behaviour of cells does not necessarily makes use of them in terms of growth [25].

### Validation: model and data consistency analysis

The datasets in Table 2 were also used to check that the experimental measurements, which reflect the metabolic state of cells, are feasible states according to the model. Two different analysis of consistency were performed: one based on minimized, variance-weighted sum of squared residuals (ϕ) and another one based on the possibility of the most possible flux state or vector (π). Both were described in the methods section. The possibilistic approach is preferred in this case because the analysis of least squares residuals has limitations due to the presence of inequality constraints in the model.

In all weighted least squares problems, a standard deviation of 10% is assigned to each measurement of the set trying to capture their uncertainty. The variance-covariance matrix **F **in (4) is defined accordingly.

In the Possibilistic MFA problems, the uncertainty of the measurements was represented as follows:

(a) Full possibility (π = 1) is assigned to values near the measured ones, less than ± 5% deviation, to account for random errors.

(b) A decreasing possibility is assigned to larger deviations so that values with a deviation equal to ± 20% have a possibility of π = 0.1 (those values with a deviation of ± 9.5% will have possibility of π = 0.5).

This representation is achieved choosing the necessary bounds (*ε*_2_^*max*^, μ_2_^*max*^) and weights (*α*, β) for each measurement *w*_*m*_. Due to (a), the bounds are defined as ε_2_^max ^= μ_2_^max ^= 0.05·*w*_*m*_. Then we operate with equations (5-7) to achieve (b). From (5) we have that, 0.2·*w*_*m *_= ε_1_^20% ^+ ε_2_^max^, and from (6) and (7), log(0.1) = -α·*ε*_1_^20%^. As a result we get that, *α *= -log(0.1)/(0.2-0.05)/*w*_*m*_. Since uncertainty is symmetric, *β *= *α*.

The results for each dataset are shown in Table 2, where the values for ϕ and π(**v_mp_**) are given. The last column provides another indicator of consistency: the degree of measurements uncertainty needed to find a flux vector in full agreement with the model constraints (π = 1). All the computations were performed with MATLAB (MathWorks Inc., 2003), and YALMIP toolbox [31] was used to conduct Possibilistic MFA.

The consistency between model and experimental measurements is very high, but for a small set. In these cases, the inconsistency pinpoints especial characteristics of these sets of data, as explained below.

The dataset D1, which corresponds to *Pichia *growing on glucose, shows very good agreement. The measured data has full possibility (π = 1), meaning that there is a flux vector compatible with model and measurements. In fact, as shown in the last column, a band of 1% around the measured values is sufficient to enclose this flux vector. Notice also that the residual is very low.

Datasets A1 and A2, which correspond to cultures growing totally or mainly on glycerol and producing a small amount of protein, also show a good agreement. The discrepancy between measurements and model is larger for A3 (π = 0.25), but still a band of 10% of deviation around measurements encloses a flux vector compatible with the model. Dataset A3 corresponds to a culture growing mainly on methanol, but supplemented on glycerol, and producing larger amounts of protein. The discrepancy is larger for A4, which corresponds to a scenario with high protein productivity.

Similar results are obtained with cultures at a higher growth rate (datasets B1-B3), B1 and B2 are highly consistent, while protein producing B3 shows similar behaviour to A3-A4. This suggests the existence of non-modelled phenomena, probably related with protein production. The agreement is quite good for the three datasets C1-C3, but the increase of the discrepancy along with higher protein expression is also noticeable.

Finally, we used two batteries of random datasets to assess whether the model is indeed able to reject flux distribution that do not correspond to actual states of *P*. *pastoris *cultures. These datasets were defined taking random combinations of values for each flux within predefined bounds (see Table 2). Most of these random scenarios were highly inconsistent with the model (possibilities lower than 0.1 in 99% and 95% of the datasets, for each battery).

In summary, the constraint-based model shows acceptable agreement with the experimental data reported by different groups for *P. pastoris *cultures, and at the same time, rejects artificially generated invalid datasets. The scenarios with lower agreement pinpoint unmodelled phenomena, possibly related to protein expression.

### Using the model to predict growth

**Figure 4:**
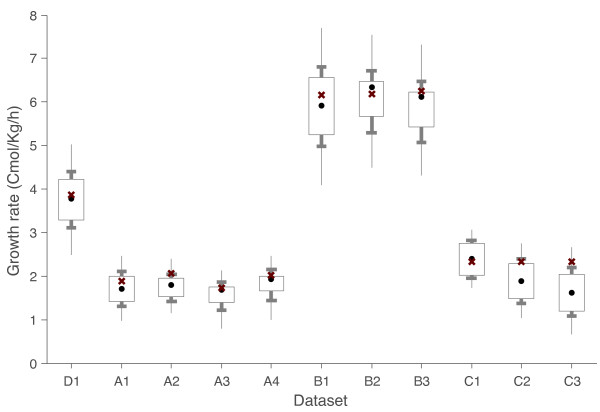
**Prediction of growth rate for *P. pastoris *cultures using Possibilistic MFA**. Crosses denote the measured values and circles most possible estimates for each dataset. The intervals of possibilities of 0.8 (box), 0.5 (bar) and 0.1 (line) are also depicted. Biomass specific growth rate is estimated as biomass efflux, expressed in Cmol·kg^-1^·h^-1^units taking into account the equivalent molecular weight of biomass provided in [19,21,22].

Possibilistic MFA can now be applied to the constraint based model and the available measurements in order to estimate the biomass growth rate for each of the previous datasets. Details of this estimation can be found in the methods section. PMFA is applied to the datasets shown above excluding the measured value of the growth rate (which is used to validate the estimation). Results are depicted in Figure 4.

The estimated growth rate is found to be in very good agreement with the measured one for the vast majority of the analysed scenarios (D1, A1, A3, A4, B1, B2, B3, C1 and C2), which correspond to cultures at different growth rates, using different substrates, and coming from three independent literature references. For two other scenarios (A2 and C3), the most possible estimate is still accurate.

The fact that, although limited, the model has predictive capacity provides further validation for this constraint-based representation. This conclusion is strengthened if we consider that the growth rate is highly interconnected along the whole network, since the biomass equation takes into account several metabolic precursors, and thus accurate correspondence between substrate uptake, respiratory fluxes and growth cannot be inferred in a straight-forward way from the network.

### Using the model to estimate the whole flux distribution

Once the model has been validated, possibilistic MFA could be used to estimate all the non-measured fluxes, either intracellular or extracellular, as done with the growth rate in the previous section. For illustration purpose, the flux distributions for each scenario are given in the additional file 3.

Notice that these estimations cannot be done by means of traditional MFA because the measurements would be insufficient to get a determined system.

**Figure 5:**
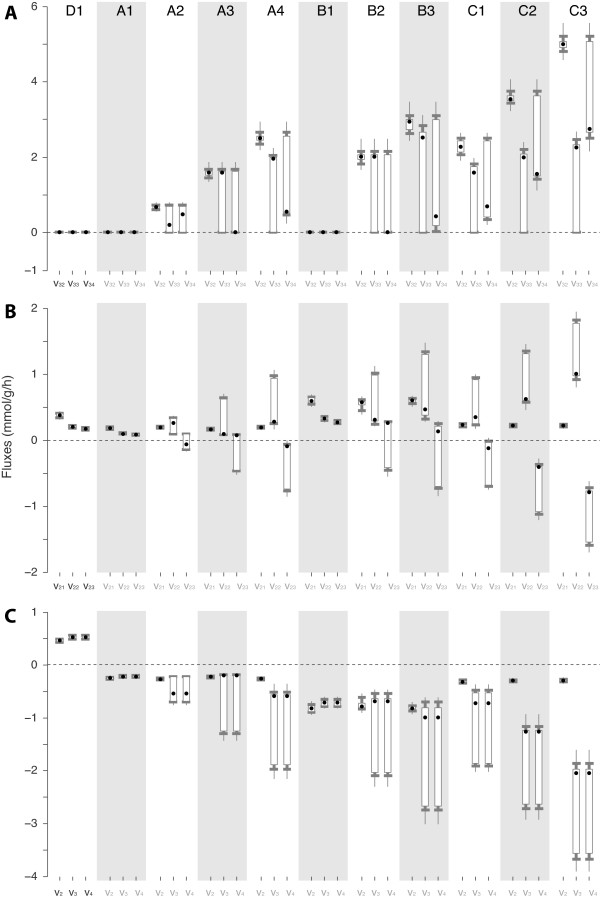
**Estimations for a set of relevant fluxes in each scenario**. Most possible values (circles and squares for measured and non measured fluxes, respectively) and intervals of conditional possibilities 0.8, 0.5 and 0.1 are depicted for each flux.

The network has 8 degrees of freedom (44 fluxes and 36 linear equations) and there are 9 measured fluxes. However, these measurements introduce only 7 independent additional linear constraints, so the system remains under-determined with 1 degree of freedom [32]. Possibilistic MFA is able to get an estimate thanks to the irreversibility constraints (other approaches considering these could also provide an estimate). Possibilistic estimates of fluxes of particular interest are also useful to perform a comparative analysis between the different scenarios and datasets. For instance, the estimates for three relevant groups of fluxes, which represent splitting nodes within the network, are depicted in Figure 5:

- Fluxes *v*_2_, *v*_3 _and *v*_4 _belonging to the glycolysis pathway, are positive as expected in cultures grown in glucose, and appear inverted in glycerol and/or methanol fed cultures.

- Fluxes *v*_21_, *v*_22 _and *v*_23 _represent the isomerization of R5P into Ru5P and Xu5P. Note how *v*_23 _inverts its direction at growing methanol fluxes, as increased methanol consumption demands higher amounts of Xu5P thus requiring more R5P precursor.

- Fluxes *v*_32_, *v*_33 _and *v*_34 _represent the branchpoint related to methanol usage, that is, how this flux is split between direct oxidation and catabolic pathways. High methanol fluxes are necessarily conducted via CO_2 _generation and thus flux *v*_34 _becomes distinct from zero in A4, B4, C2 and C3 scenarios.

In this way, these results further validate the predictive capability of the model.

## Conclusions

The consistency of a constraint-based model of *Pichia pastoris *has been validated in several experimental scenarios resulting in good agreement between estimations and measurements. In addition, the predictive capacity of the model for cell growth rate, an attractive target for industrial fermentation monitoring and control, has been verified. Interestingly, the accuracy of predictions worsens for higher protein producing scenarios, showing how the model, derived for a wild-type strain, is increasingly less precise as wider resources are devoted to recombinant protein generation.

It must be highlighted that the model has been strictly constructed upon first-principles and sensible hypothesis. At this point, the model can be curated, extended, and its parameters tuned in order to improve the consistency with the investigated scenarios. Particularly, energy requirements, strongly related to protein expression, are not yet considered within the model and future work will address this issue.

This contribution shows how a small-sized network can in general be assessed following a rational, quantitative procedure even when measurements are scarce. Possibilistic MFA becomes a useful tool to systematize this procedure. This approach enables validation considering the stoichiometric balances and also reactions reversibilities, and accounting for measurements imprecision. The use of Possibilistic MFA also makes it possible to predict non-measured fluxes without removing the network under-determinancy. There is, however, a challenge when validating networks with higher number of degrees of freedom because there may be many flux vectors compatible with the (few) available measurements. It is expected that the datasets will be highly consistent, so the approach in this case would be to check if the model rejects the artificially generated invalid datasets.

When a validated model is available, ideally incorporating measurements for some intracellular fluxes, the kind of comparative analysis proposed herein will provide a insight on how the internal state of the cells determines its external behavior, and potentially lead intervention within cells, suggesting target metabolites or biochemical branch-points and also allowing optimization through manipulation of extracellular variables, such as feeding strategies and substrate selection.

## Authors' contributions

MTS, FLL and JPM designed the research and conceptualized the manuscript. MTS elaborated the metabolic network; FLL designed the consistency analysis method. MTS and FLL analyzed the results and drafted the manuscript. JPM supervised and coordinated the project. All authors read and approved the final manuscript.

## Acknowledgements

This research has been partially supported by the Spanish Government (2^nd ^and 3^rd ^authors are grateful to grants DPI2008-06880-C03-01 and A/016560/08). FLL is recipient of a fellowship from the Spanish Ministry of Science and Innovation (FPU AP2005-1442). The authors are grateful to the Company Biopolis for his support to this research.

## Supplementary Material

Additional file 1 **Metabolic network for *P. pastoris***. This includes the list of reactions, metabolites and stoichiometric matrix. Click here for file

Additional file 2 **Elementary mode analysis**. This file includes the whole set of elementary modes, the corresponding macroreactions and the calculation of the theoretical yields. Click here for file

Additional file 3 **Complete flux distribution per scenario**. This file includes the figures representing the estimation of each intracellular flux for all datasets. Click here for file
