# Supplementary material for: Validation of a constraint-based model of Pichia pastoris metabolism under data scarcity
Source: BMC Syst Biol. 2010 Aug 17;4:115. doi: 10.1186/1752-0509-4-115 (PMC2936294; doi:10.1186/1752-0509-4-115)
Supplement: Additional file 3 — Complete flux distribution per scenario. This file includes the figures representing the estimation of each intracellular flux for all datasets. [file 1752-0509-4-115-S3.PDF]

Additional file 4: Complete flux distribution per each scenario

Marta Tortajada*, Francisco Llaneras** and Jesús Picó**

* Biopolis S.L., Dept. of Microbial Biotechnology, Valencia, Spain.

** Instituto de Automática e Informática Industrial, Universidad Politécnica de Valencia, Spain.

Corresponding author: Francisco Llaneras, {frallaes@upvnet.upv.es; kikollan@gmail.com}

Scenario D1

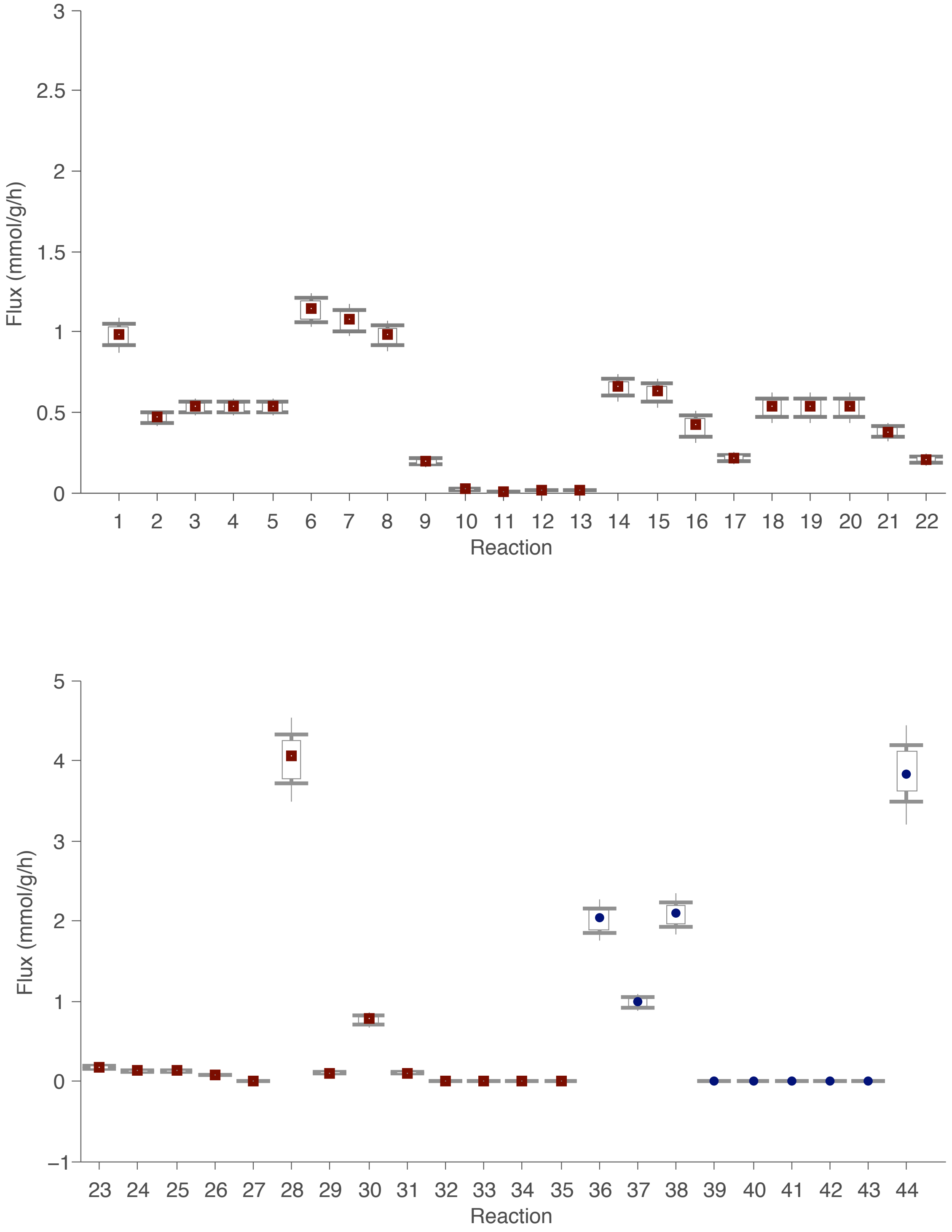

Figure. Flux estimation performed with Possibilistic MFA. Most possible values (circles and squares for measured and non measured fluxes, respectively) and intervals of conditional possibility 0.8, 0.5 and 0.1 are depicted for each flux/reaction.

Scenarios A1

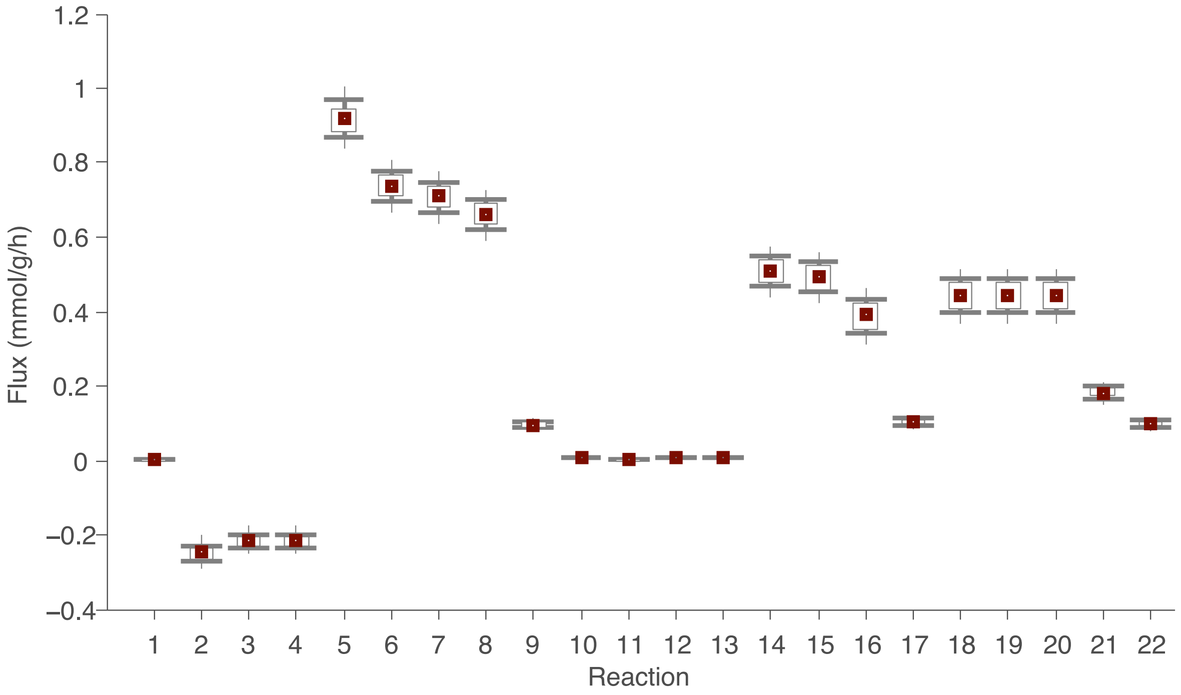

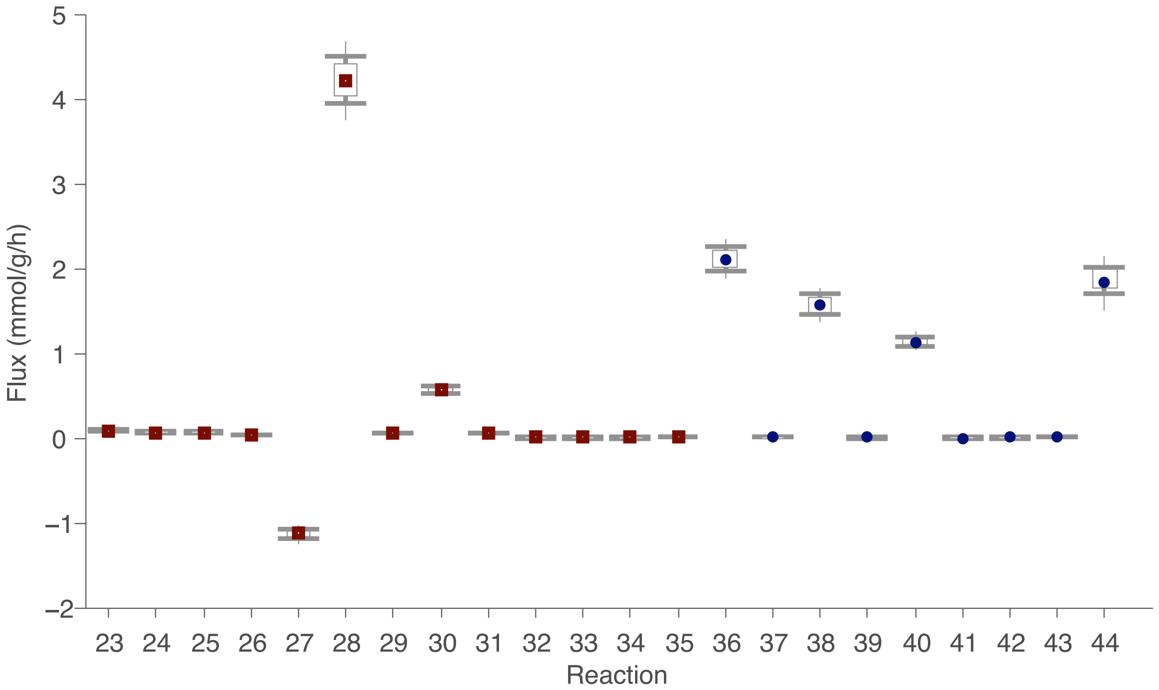

Figure. Flux estimation performed with Possibilistic MFA. Most possible values (circles and squares for measured and non measured fluxes, respectively) and intervals of conditional possibility 0.8, 0.5 and 0.1 are depicted for each flux/reaction.

Scenarios A2

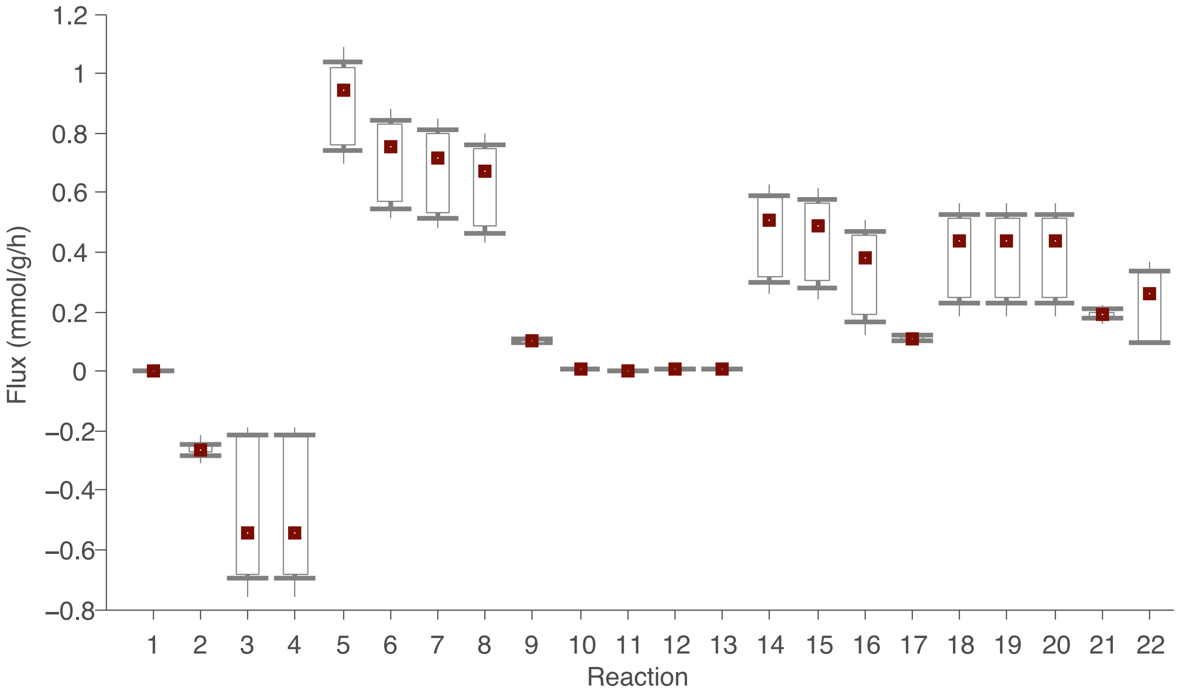

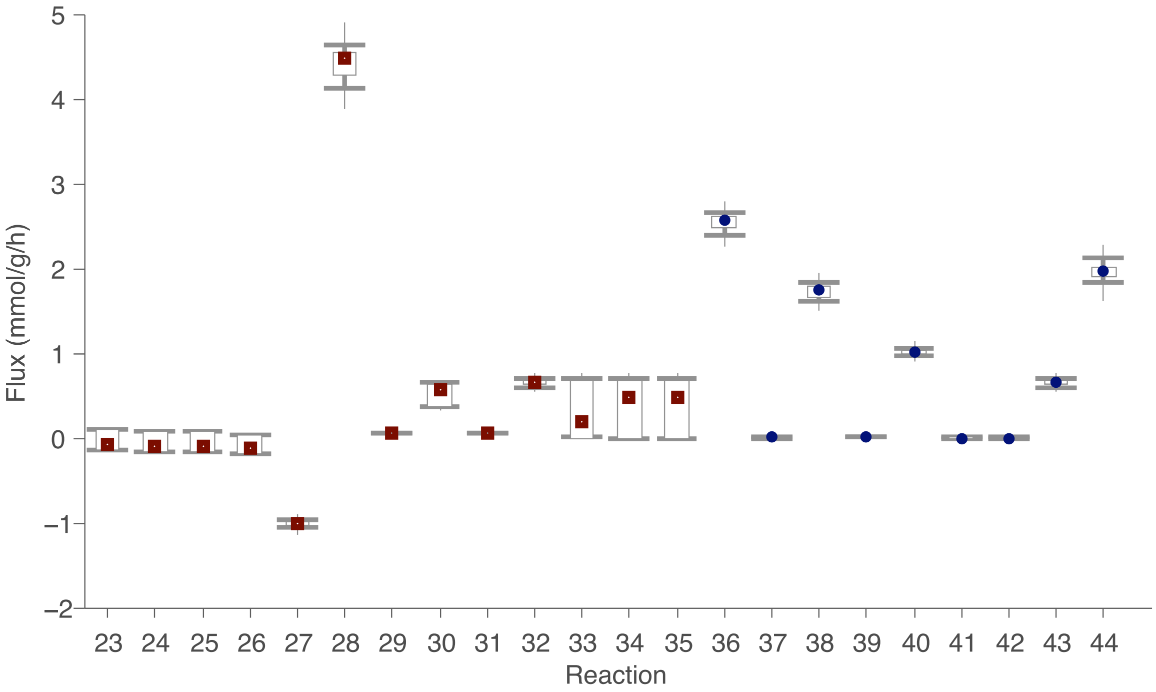

Figure. Flux estimation performed with Possibilistic MFA. Most possible values (circles and squares for measured and non measured fluxes, respectively) and intervals of conditional possibility 0.8, 0.5 and 0.1 are depicted for each flux/reaction.

Scenarios A3

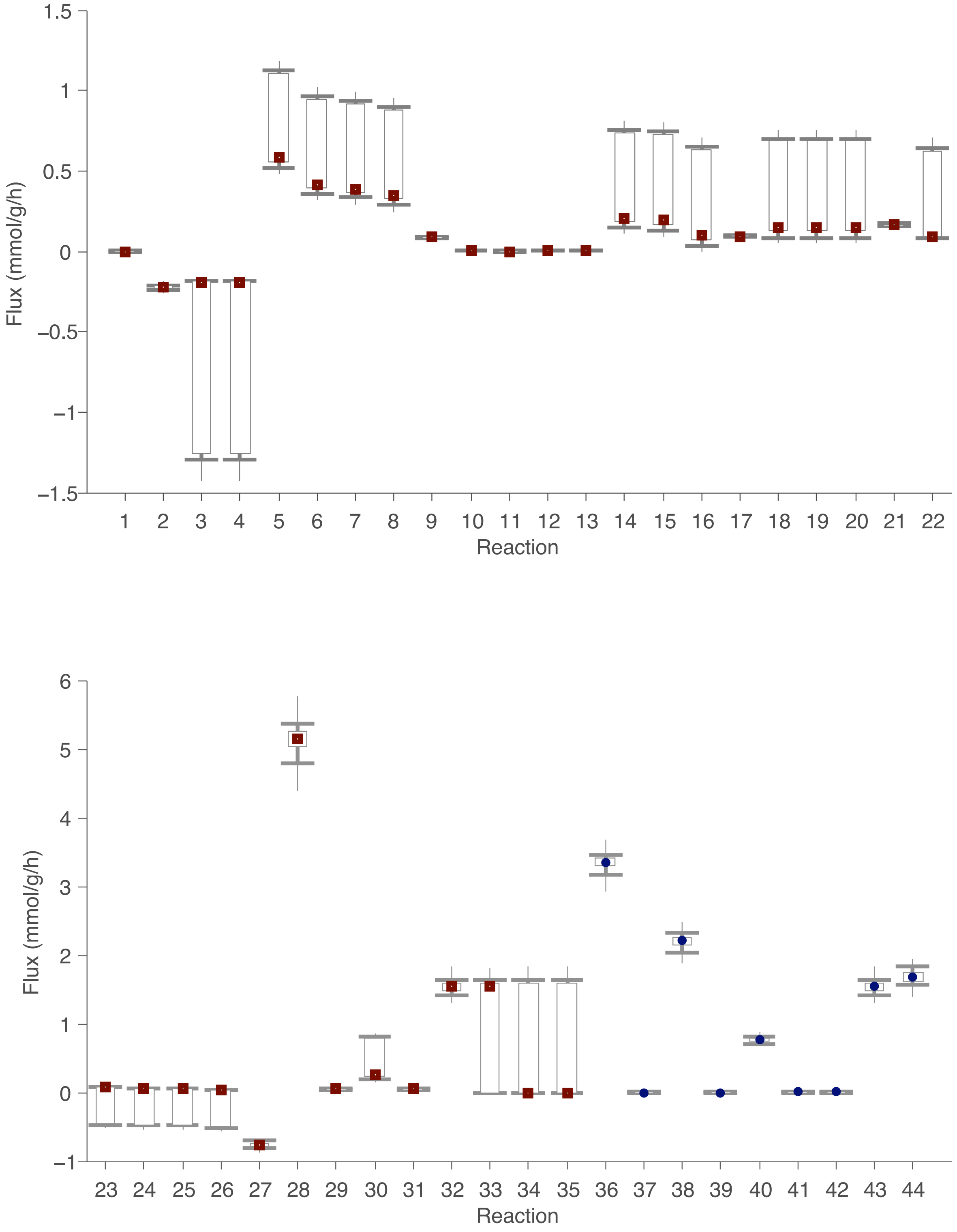

Figure. Flux estimation performed with Possibilistic MFA. Most possible values (circles and squares for measured and non measured fluxes, respectively) and intervals of conditional possibility 0.8, 0.5 and 0.1 are depicted for each flux/reaction.

Scenarios A4

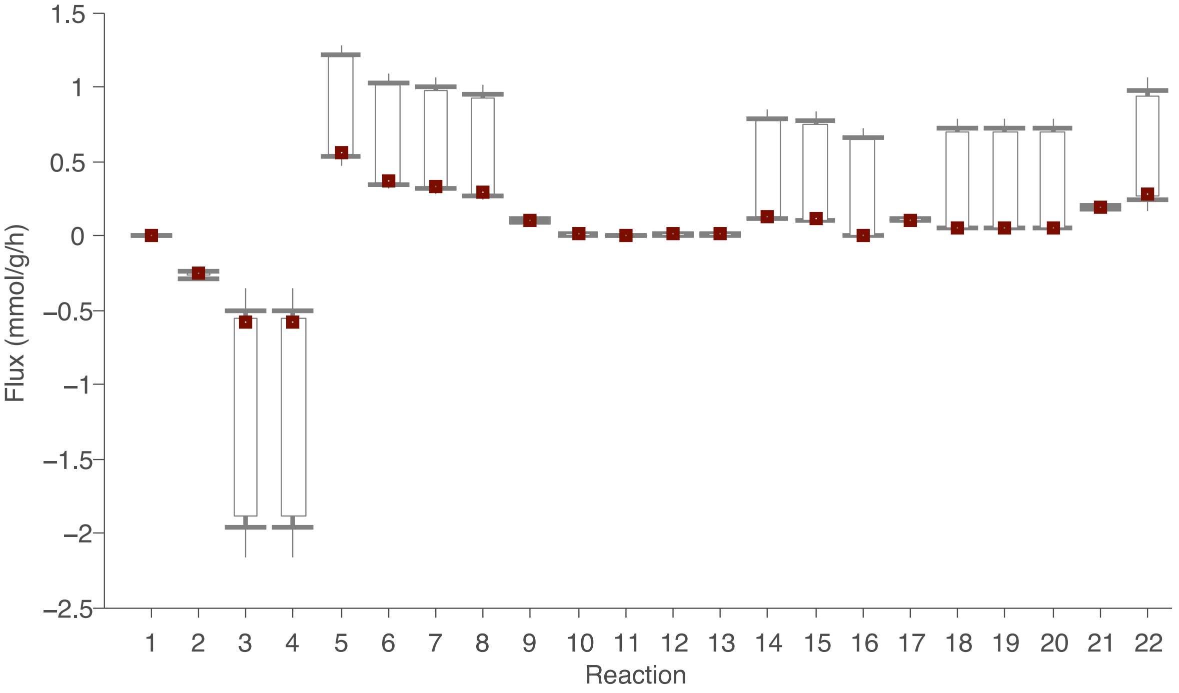

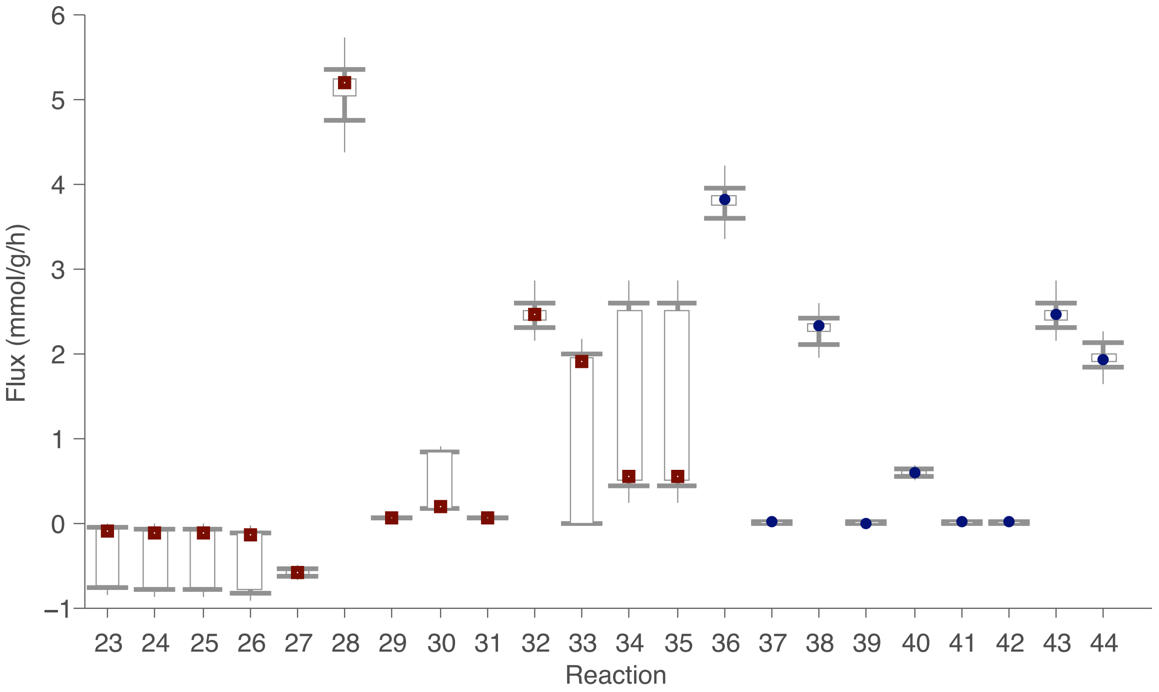

Figure. Flux estimation performed with Possibilistic MFA. Most possible values (circles and squares for measured and non measured fluxes, respectively) and intervals of conditional possibility 0.8, 0.5 and 0.1 are depicted for each flux/reaction.

Scenarios B1

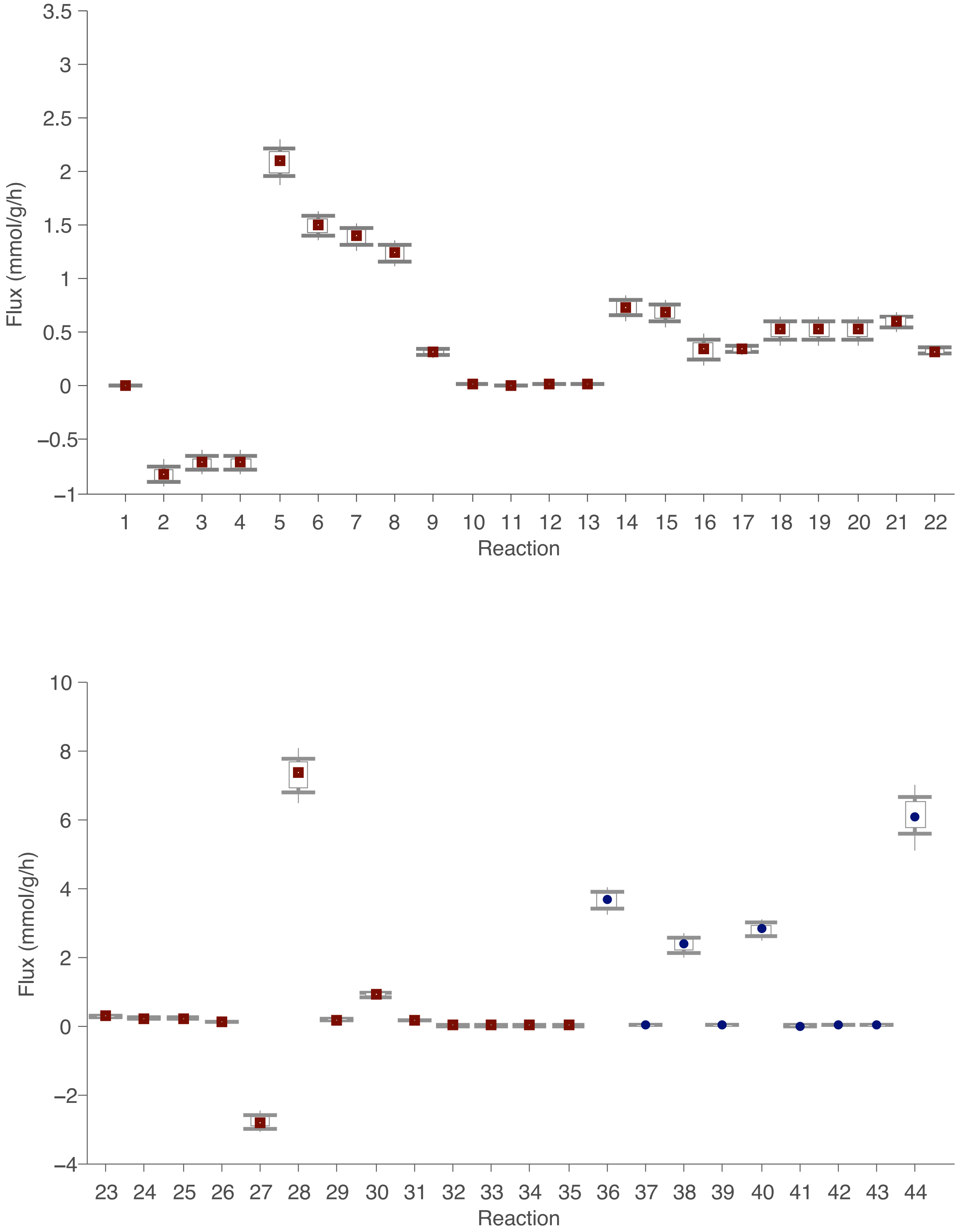

Figure. Flux estimation performed with Possibilistic MFA. Most possible values (circles and squares for measured and non measured fluxes, respectively) and intervals of conditional possibility 0.8, 0.5 and 0.1 are depicted for each flux/reaction.

Scenarios B2

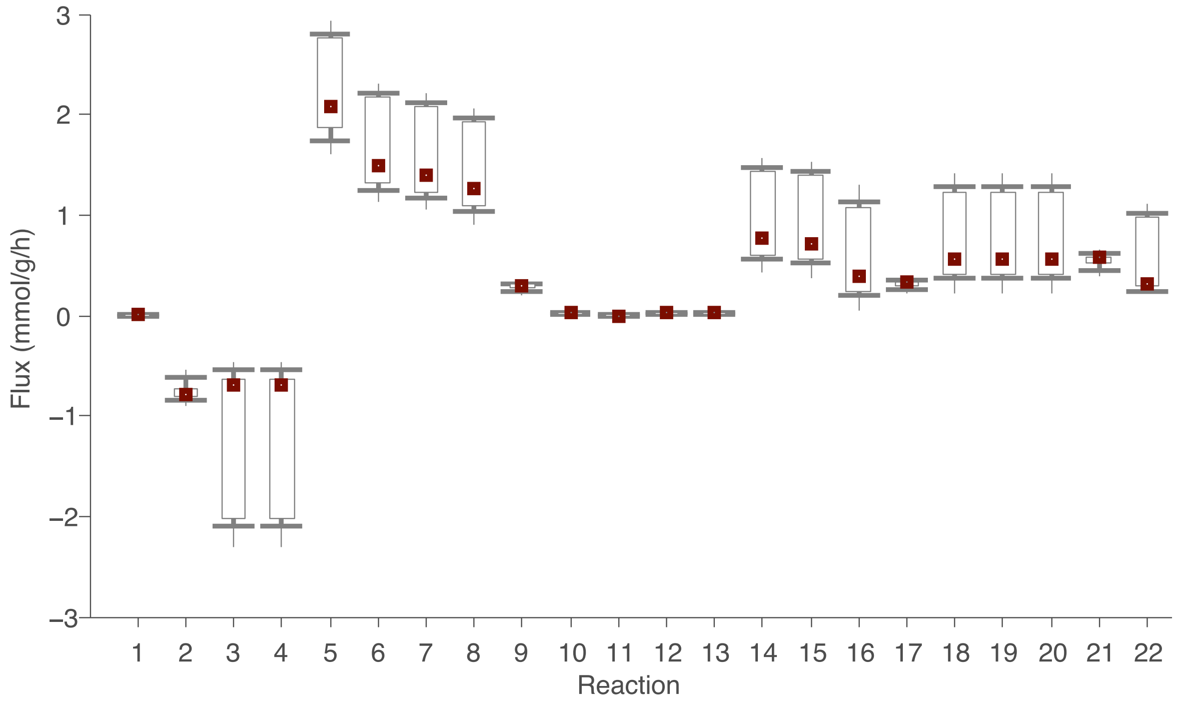

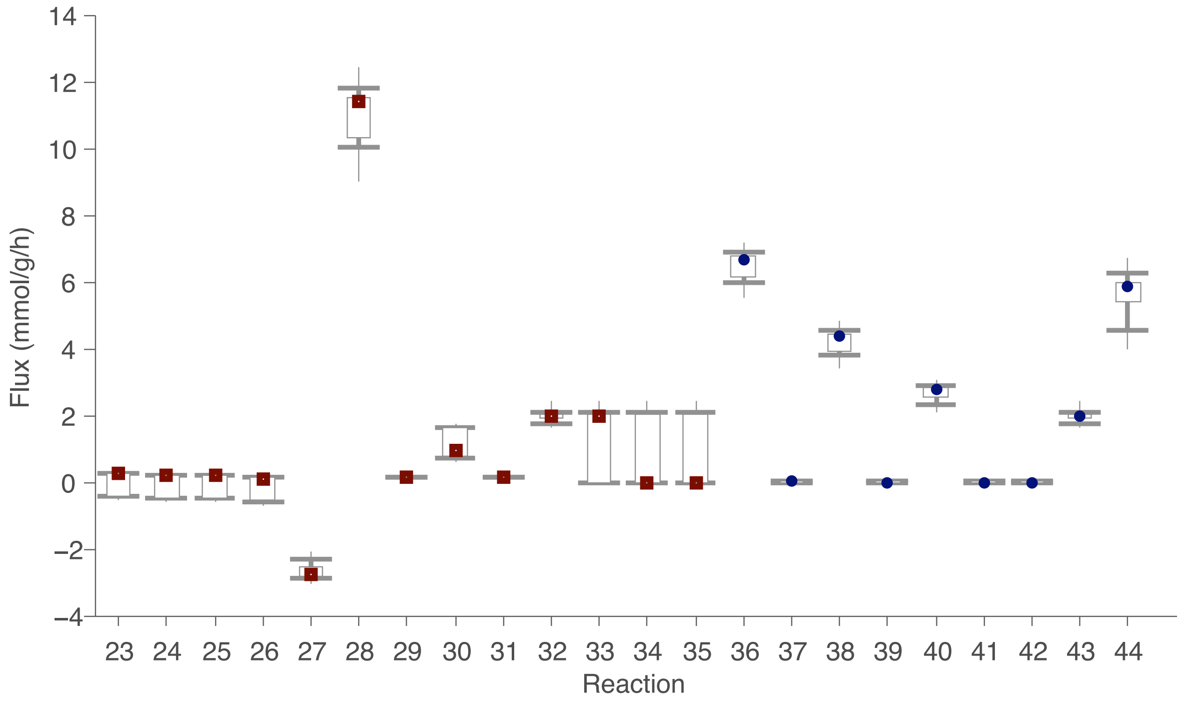

Figure. Flux estimation performed with Possibilistic MFA. Most possible values (circles and squares for measured and non measured fluxes, respectively) and intervals of conditional possibility 0.8, 0.5 and 0.1 are depicted for each flux/reaction.

Scenarios B3

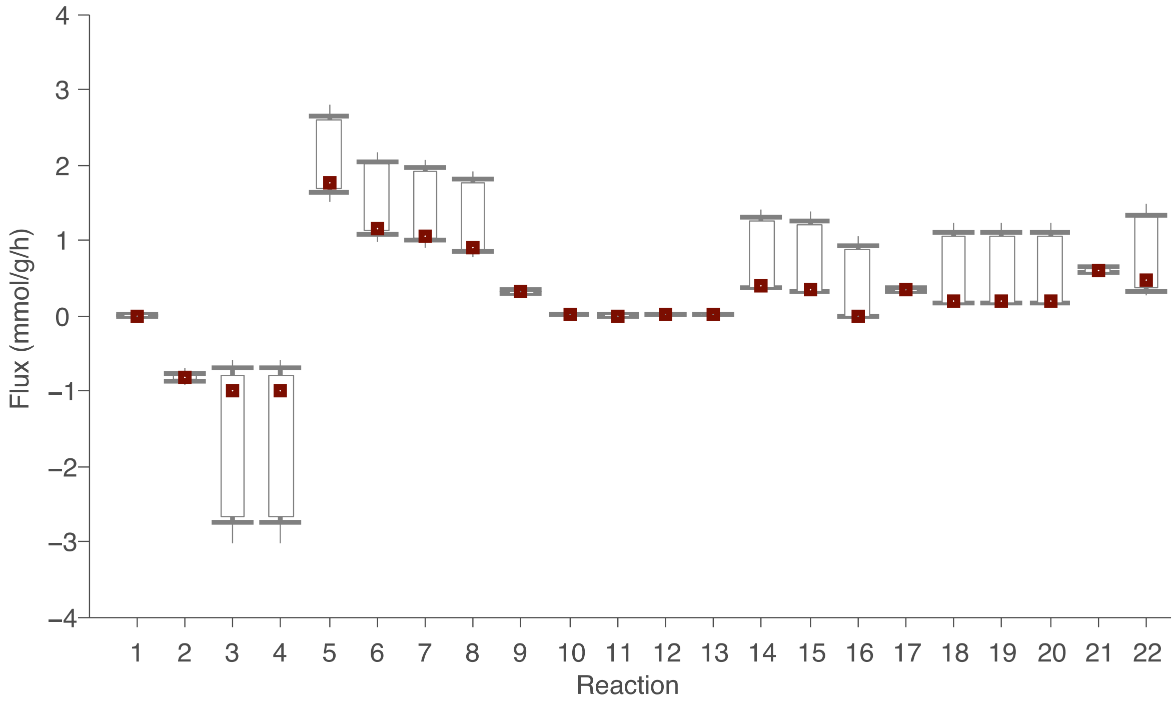

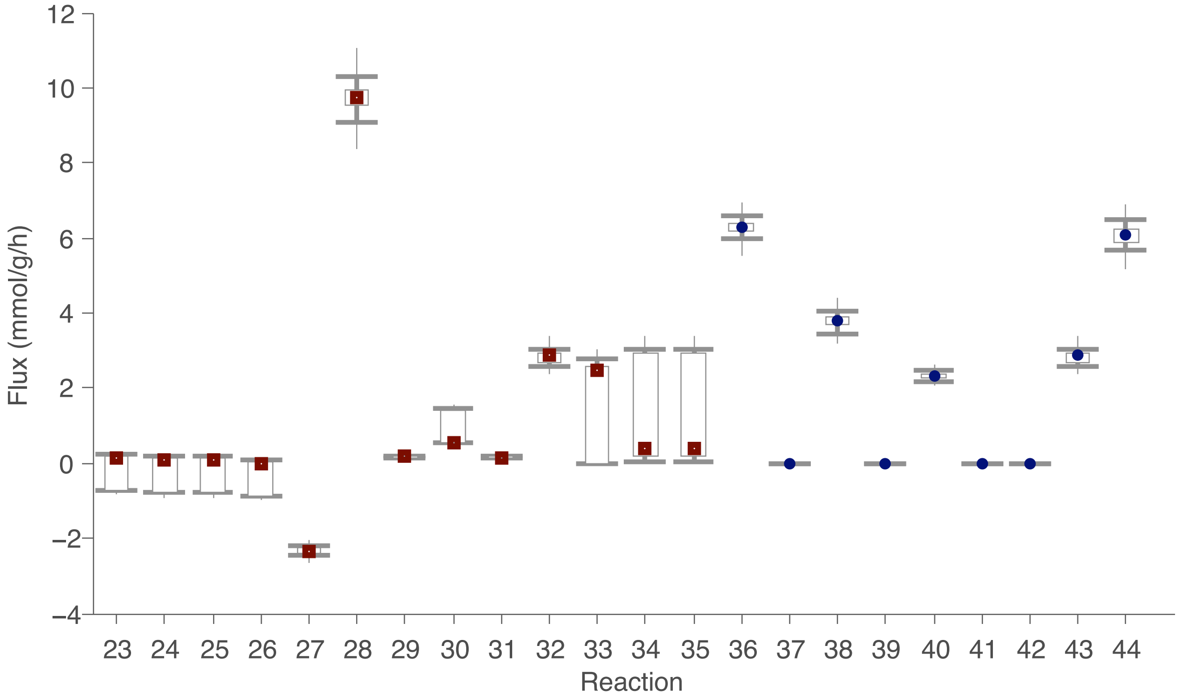

Figure. Flux estimation performed with Possibilistic MFA. Most possible values (circles and squares for measured and non measured fluxes, respectively) and intervals of conditional possibility 0.8, 0.5 and 0.1 are depicted for each flux/reaction.

Scenarios C1

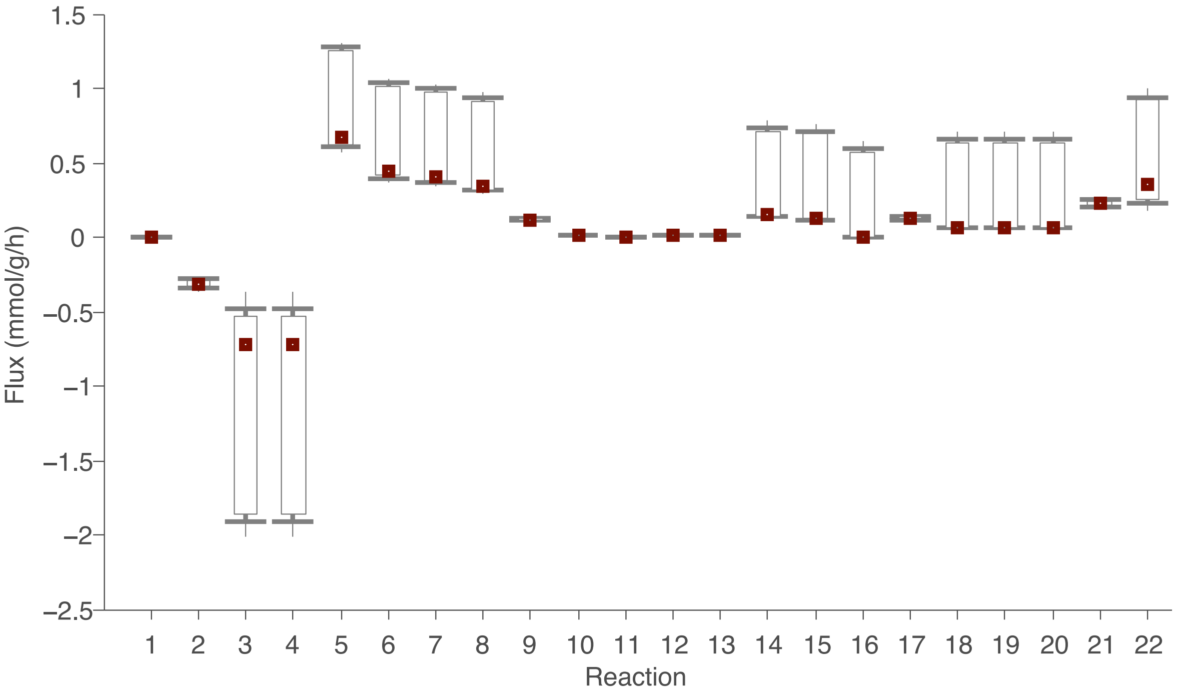

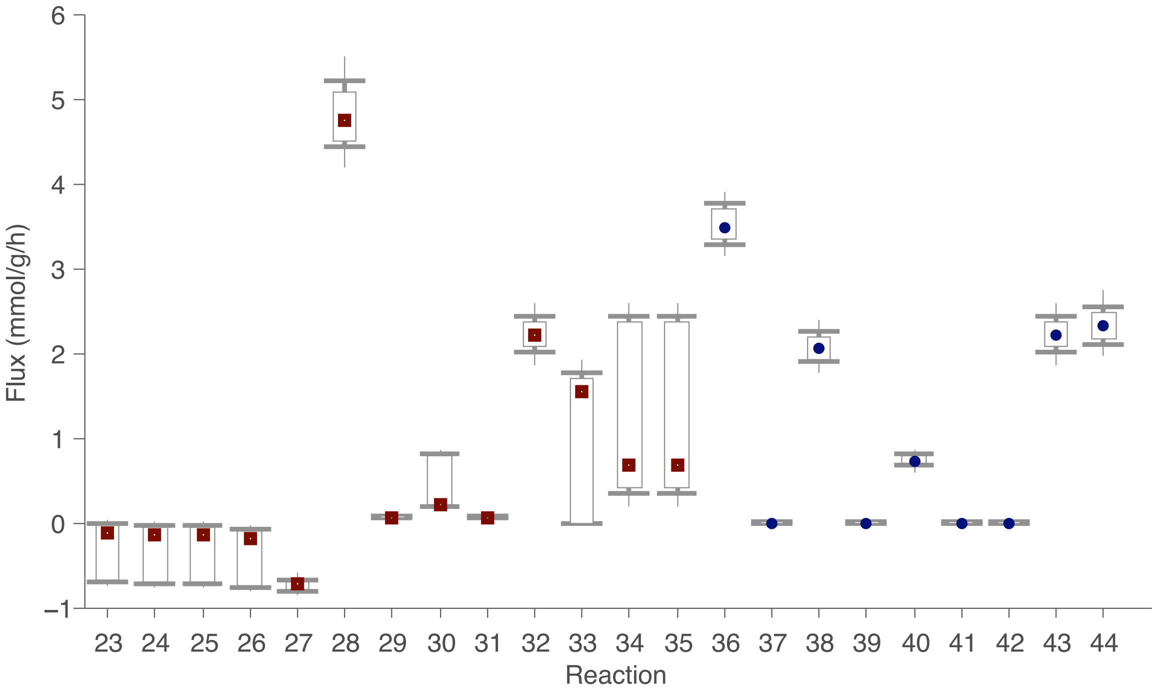

Figure. Flux estimation performed with Possibilistic MFA. Most possible values (circles and squares for measured and non measured fluxes, respectively) and intervals of conditional possibility 0.8, 0.5 and 0.1 are depicted for each flux/reaction.

Scenarios C2

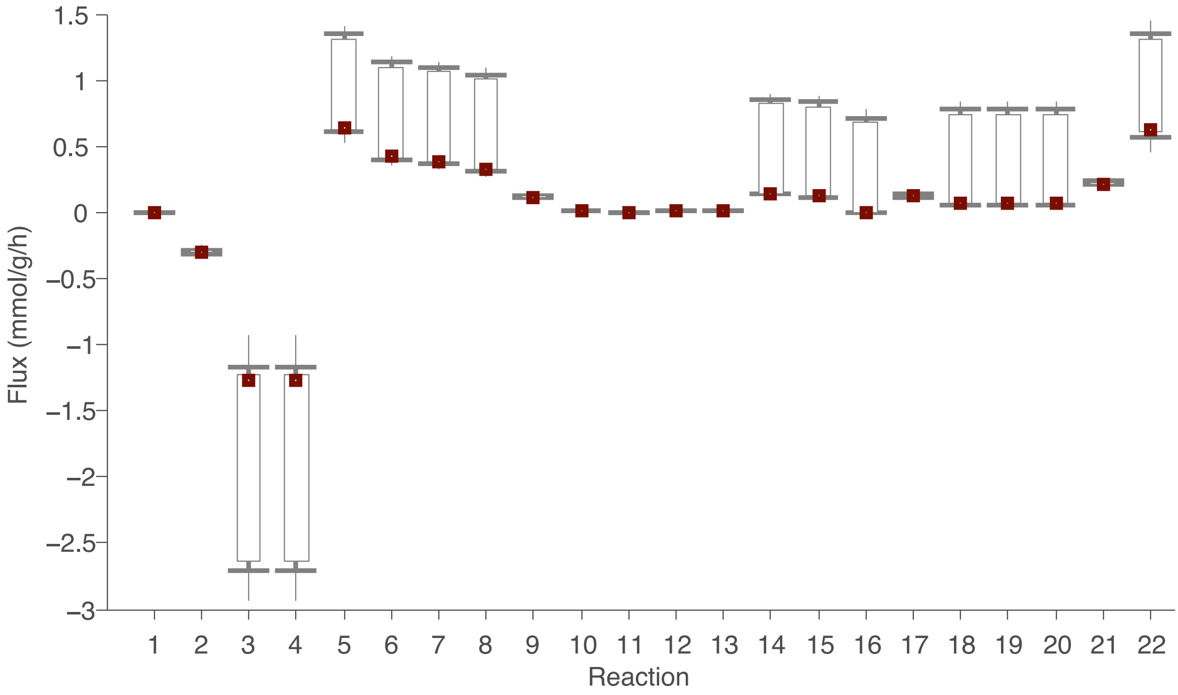

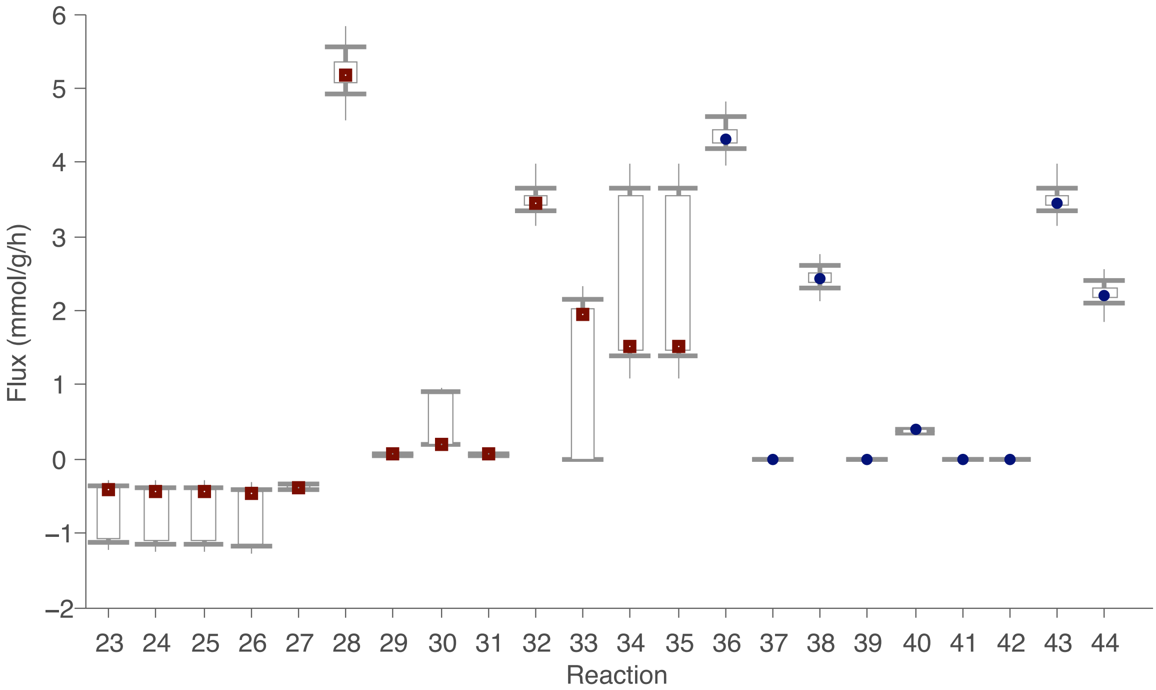

Figure. Flux estimation performed with Possibilistic MFA. Most possible values (circles and squares for measured and non measured fluxes, respectively) and intervals of conditional possibility 0.8, 0.5 and 0.1 are depicted for each flux/reaction.

Scenarios C3

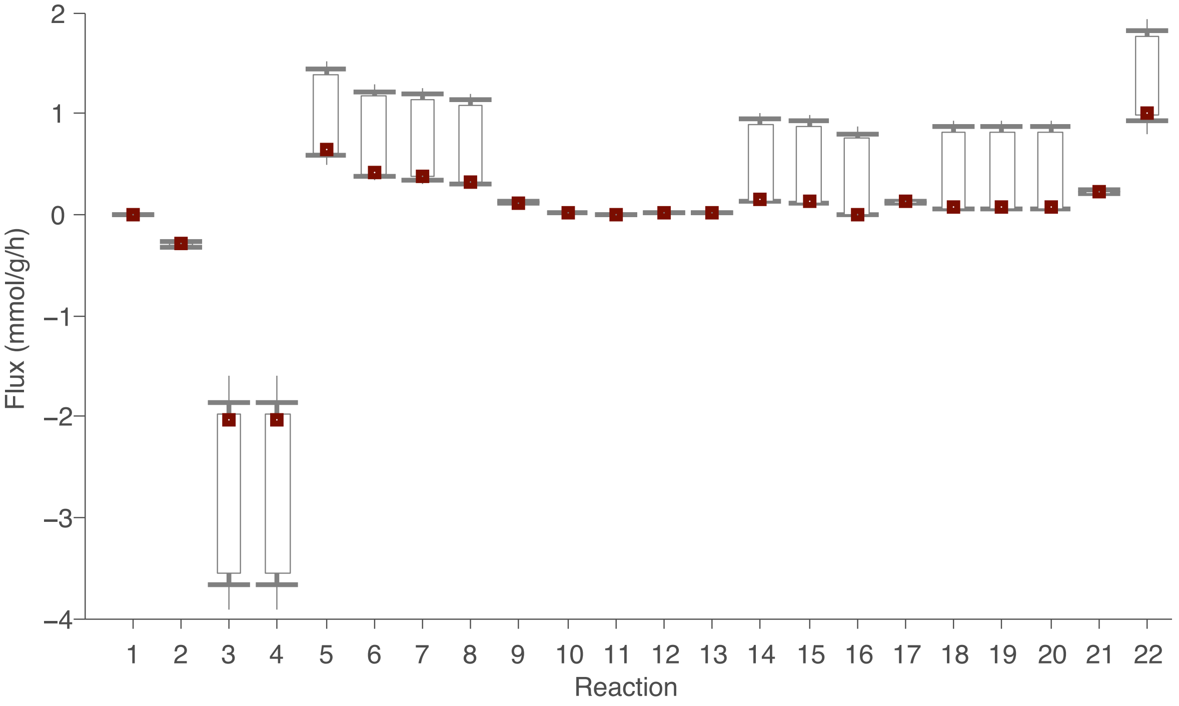

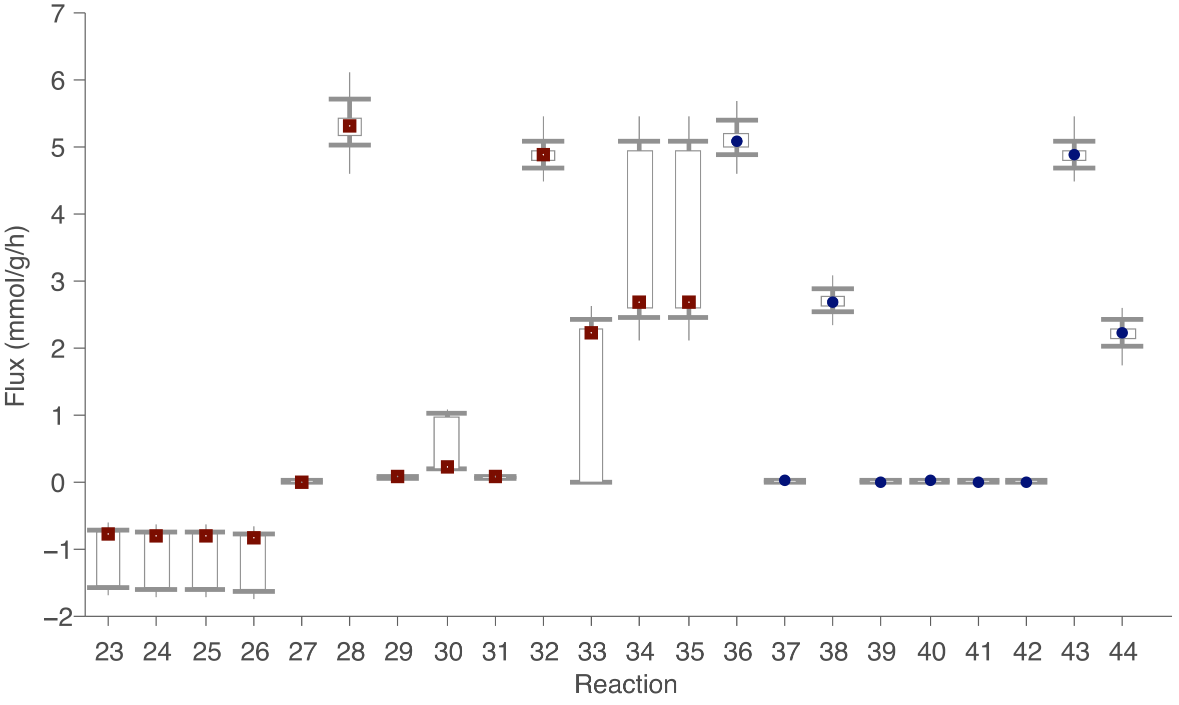

Figure. Flux estimation performed with Possibilistic MFA. Most possible values (circles and squares for measured and non measured fluxes, respectively) and intervals of conditional possibility 0.8, 0.5 and 0.1 are depicted for each flux/reaction.
